# Diagnostic Accuracy of Intraoral, Extraoral and Cone Beam Computed Tomography (CBCT)-Generated Bitewings for Detecting Approximal Caries and Periodontal Bone Loss

**DOI:** 10.7759/cureus.95529

**Published:** 2025-10-27

**Authors:** Jyoti Mago, Alan G Lurie, Aadarsh Gopalakrishna, Aditya Tadinada

**Affiliations:** 1 Oral and Maxillofacial Radiology, University of Nevada, Las Vegas, Las Vegas, USA; 2 Oral and Maxillofacial Radiology, University of Connecticut, Farmington, USA; 3 Operative Dentistry, University of Connecticut, Farmington, USA

**Keywords:** 180-degree rotation cbct scans, 360-degree rotation cbct scans, approximal caries, low dose cbct, periodontal bone loss, two-wall defect

## Abstract

Purpose: This study aimed to evaluate the diagnostic accuracy of intraoral bitewings (IB), extraoral bitewings (EB) generated from panoramic radiographs and cone beam computed tomography (CBCT) scans, using both 180° and 360° rotation protocols, for detecting approximal dental caries and periodontal bone loss.

Methodology: Fifty human teeth accounting for one hundred interproximal surfaces in three partially/fully dentate dry human mandibles were evaluated for carious lesions, alveolar bone levels, and interdental vertical bone defects in the posterior region. Intraoral bitewings were acquired using photostimulable phosphor (PSP) sensors with a KaVo LM/CM109 X-ray tube (70 kVp, 7 mA, 0.125 s; KaVo, Charlotte, NC, USA). Extraoral bitewings were obtained using a J. Morita panoramic unit (90 kVp, 7 mA, 5.5 s; J. Morita, Irvine, CA, USA). CBCT scans were performed with a J. Morita 3D Accuitomo system (90 kVp, 7 mA, 40×40 mm field of view (FOV), Hi-Res Mode) under both 180° and 360° rotation protocols. A single board-certified oral and maxillofacial radiologist assessed the presence and severity of caries and conducted linear measurements of alveolar bone levels. Diagnostic findings were compared across imaging modalities.

Results: No statistically significant differences were observed among the intraoral, extraoral, and CBCT imaging modalities regarding the detection of carious lesions and linear measurements of periodontal bone loss.

Conclusion: Extraoral bitewings generated from panoramic radiographs and CBCT (180° and 360°) scans demonstrated diagnostic accuracy comparable to intraoral bitewings for the detection of approximal caries and periodontal bone defects.

## Introduction

The current screening protocol for radiographic evaluation recommended by the American Dental Association (ADA) includes a bitewing radiograph and a panoramic radiograph to detect interproximal caries and bone loss.

Traditionally, interproximal caries has been detected radiographically using intraoral bitewing (IB) images [1,2]. While this has been the conventional practice, the challenge of accommodating the image receptor in the mouth especially in patients with gag reflex has affected image acquisition and quality. Hence, newer modalities such as extraoral bitewing (EB) radiography using a panoramic machine and cone-beam computed tomography (CBCT)-generated bitewings are now in the arena of research for their diagnostic efficacy and dosimetry [3-7].

With COVID-19, the use of extraoral radiography will additionally reduce salivary contamination and will reduce the use of personal protective equipment as much information can be gained with one acquisition.

With the advent of CBCT in dental radiography, detecting its diagnostic accuracy for dental caries and periodontal bone loss was a logical challenge. With the current-generation CBCT scanners, the option of image acquisition with only a 180-degree rotation CBCT scan has become available. This reduced-dose option makes CBCT a viable consideration for many more dental applications that previously did not use 3-D imaging due to dose considerations. The use of 180-degree rotation also reduces the exposure time and reduces the basis image. However, the diagnostic accuracy of 180-degree rotation in CBCT scans has not been studied widely until now due to its recent introduction, and not all machines have this capability.

Keeping these factors in mind, the hypothesis was made that CBCT at both 180-degree and 360-degree rotations has a superior diagnostic efficacy for diagnosing approximal caries and bone loss as compared to intraoral bitewings and extraoral bitewings and the diagnostic efficacy of these modalities were compared.

This article was previously presented as a meeting abstract at the virtual American Academy of Oral and Maxillofacial Radiology (AAOMR) Annual Scientific Meeting held in Oct., 2020.

## Materials and methods

**Figure 1:**
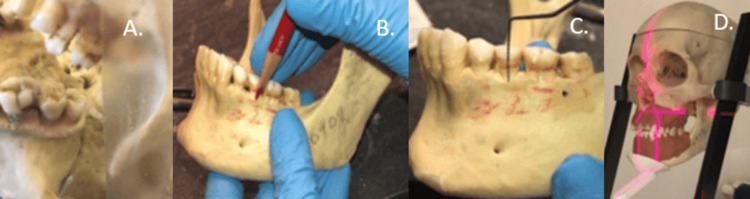
A. Simulated carious defect, B. Site marked prior to bone loss creation, C. Simulated bone loss defect, D. Image acquisition protocol

Fifty human teeth accounting for one hundred interproximal surfaces in three partially/fully dentate dry skulls were used in this study. The current project examined carious defects, alveolar bone levels and interdental vertical defects in the posterior mandible in an ex vivo model for bitewings. Each mandible was assessed visually to determine the presence of molar and premolar teeth (Figure 1).

Mandibles that were edentulous, contained only anterior mandibular teeth, had teeth fractured to the level of the alveolar crest, or teeth containing any metallic restorations, were excluded from the study. Furthermore, teeth with overlapping contacts or where all surfaces of the tooth were not seen on the images were also excluded from the study.

Carious lesions

Carious lesions were simulated using a round diamond bur by a research associate (L.E.), who was not involved in the scoring of the images. The simulated defects were randomly assigned across the enamel, dentin, and pulp on the interproximal surfaces. The grading criteria for these lesions are summarized in Table 1.

**Table 1:** Grading of Carious Lesions

Grade of carious lesions	Extent
0	No caries
1	Caries into enamel but not to dentinoenamel junction (DEJ)
2	Caries into dentin but not involving the pulp
3	Caries involving pulp

Periodontal evaluation

For soft-tissue simulation, the maxilla and mandible were covered by a layer of impression compound. A periodontal consultant (A.M.) randomly created 50 periodontal bone defects at depths of 2 mm, 4 mm, or 6 mm on all four tooth surfaces - buccal, lingual, mesial, and distal. Additionally, two one-wall defects and six two-wall defects were also created interdentally in the posterior sextants of the maxilla and the mandible keeping four sites with the buccal wall intact and four with lingual walls intact.

All osseous defects were measured directly on the dry skulls using a periodontal probe. A periodontist (A.M.) documented the presence, type, and location of each defect, which were recorded in a datasheet and later served as the gold standard for comparison. The grading criteria for periodontal lesions are presented in Table 2.

**Table 2:** Grading of the periodontal lesions

Grade of periodontal lesions	Periodontal lesions
0	Present
1	Absent
2	Uncertain

Image acquisition

**Figure 2:**
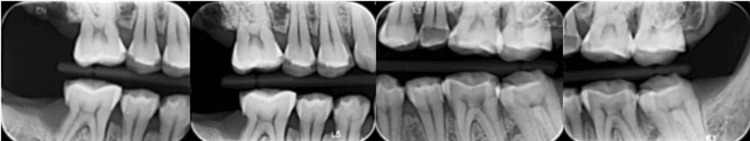
Intraoral radiographs

Intraoral bitewings were acquired using photostimulable phosphor (PSP) plates and a KaVo (Charlotte, NC, USA) LM/CM109 X-ray unit (70 kilovoltage peak (kVp), 7 milliamperes (mA), 0.125 s) with circular collimation (Figure 2).

**Figure 3:**
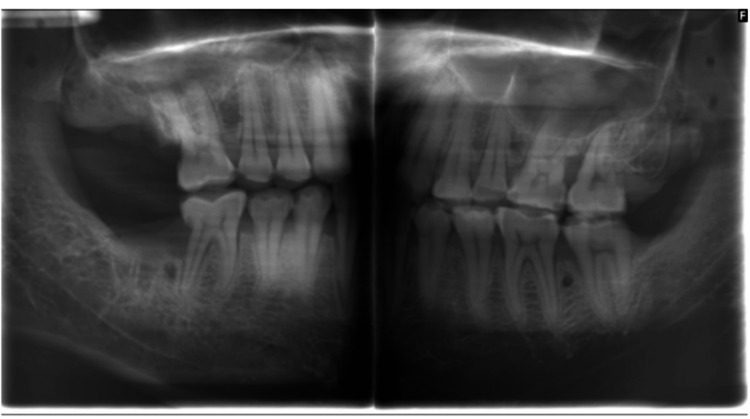
Extraoral radiograph

Extraoral bitewings were taken with a J. Morita (Irvine, CA, USA) unit (90 kVp, 7 mA, 5.5 s) (Figure 3).

**Figure 4:**
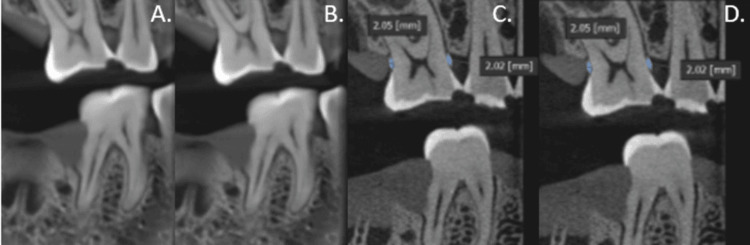
Cone beam computed tomography (CBCT) images showing carious defects at 180° (A) and 360° (B); bone loss measurements at 180° (C) and 360° (D) rotations, respectively.

CBCT scans were obtained using the J. Morita 3D Accuitomo (90 kVp, 7 mA, 40 × 40 mm field of view (FOV)) in high-resolution mode. Axial and multiplanar reconstructions were generated, and volumetric data were used to create serial coronal and sagittal sections along each tooth plane (Figure 4).

Image display

Images were evaluated using MiPACS software (Medical Imaging, Charlotte, NC, USA) on dual standard radiology-grade display monitors.

Image evaluation

Each image set was independently and randomly evaluated by a trained oral and maxillofacial radiologist using Xelis Dental software (INFINITT Healthcare Co., Seoul, South Korea). The examiner was blinded to the defects and assessed carious and periodontal lesions in each modality, including both CBCT rotation angles, based on the predefined grading criteria.

For periodontal evaluation, measurements were performed using the distance measurement tool in MiPACS for intraoral and extraoral images, and in Xelis for CBCT volumes. Interdental vertical defects were identified when the alveolar crest was not parallel to the line connecting the cementoenamel junctions (CEJs) of adjacent teeth. The number of remaining osseous walls was assessed on CBCT in both 180- and 360-degree rotation bitewings.

In CBCT scans (180° and 360° rotations), defect depth was measured in three planes: 1) superior-inferiorly from the line joining CEJ’s of adjacent teeth till the alveolar crest and was noted in the sagittal section, 2) mesiodistally from the proximal surface of the tooth till the maximum dimension of the defect in the mesiodistal direction, which was noted in the axial slice, and 3) buccolingually from the level of surrounding alveolar crest buccally/lingually to the maximum dimension of the defect which was also viewed in the axial slice.

**Figure 5:**
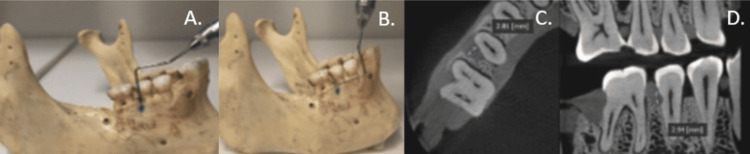
Measurement of two-wall defect. A. Superior-inferior measurement (dry mandible), B. Mesiodistal measurement (dry mandible), C. Mesiodistal measurement (cone beam computed tomography (CBCT)), D. Superior-inferior measurement (CBCT)

Measurements were done both physically on the dry human skull and CBCT with both 180-degree and 360-degree rotations (Figure 5). These values were later compared by statistical evaluation.

Statistical analysis

The statistical analysis was carried out using Statistical Package for Social Sciences (SPSS, version 15.0; SPSS Inc., Chicago, IL, USA). Quantitative variables were summarized using measures of central tendency (mean, median) and measures of dispersion (standard deviation). The Wilcoxon Signed-Rank test was performed for comparison of groups for simulated carious defects and simulated periodontal bone loss. A p-value of less than 0.01 was considered statistically significant for the measurement of periodontal bone loss.

The Friedman test was applied to assess differences in carious lesion detection across imaging modalities. Paired t-tests were used to compare measurements of osseous wall defects. A significance level of p < 0.05 was used for all other comparisons.

## Results

**Figure 6:**
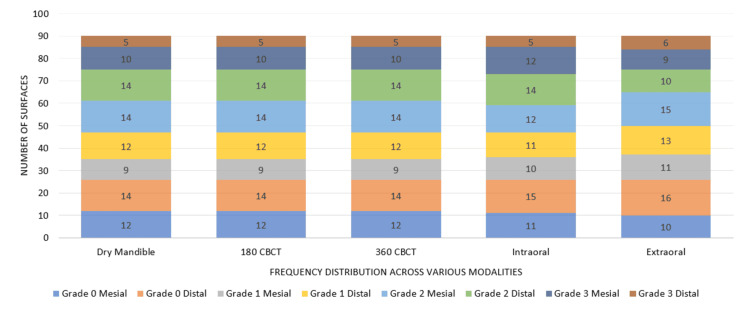
Frequency (n) of Simulated Carious Defects Across Imaging Modalities by Caries Severity Grade CBCT: cone beam computed tomography

Based on the exclusion criteria, 45 of 50 teeth (90 proximal surfaces) were included for evaluating simulated carious and periodontal defects. Out of these, 33 carious lesions were located on the mesial surfaces (nine enamel, 14 dentin, and 10 pulpal), and 31 on the distal surfaces (12 enamel, 14 dentin, and five pulpal). The detection of these carious lesions across various imaging modalities, based on the predefined grading criteria, is summarized below (Figure 6).

**Figure 7:**
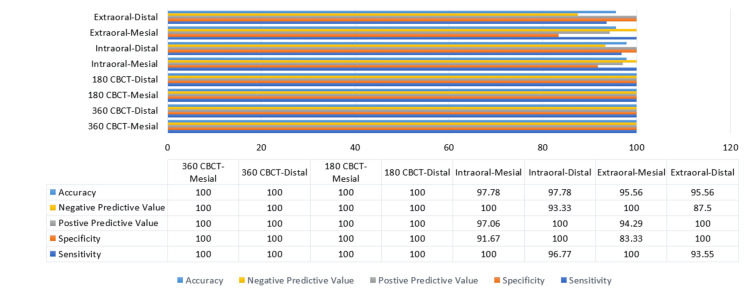
Intergroup Statistical Comparison of Simulated Carious Defects CBCT: cone beam computed tomography

When compared to the simulated defects on the dry skull, both 180-degree and 360-degree CBCT rotations demonstrated identical performance, achieving 100% accuracy, sensitivity, specificity, positive predictive value, and negative predictive value on both mesial and distal surfaces (Figure 7). Overall, extraoral bitewings showed the lowest diagnostic performance in detecting the presence or absence of defects; however, their results remained comparable to other modalities.

**Figure 8:**
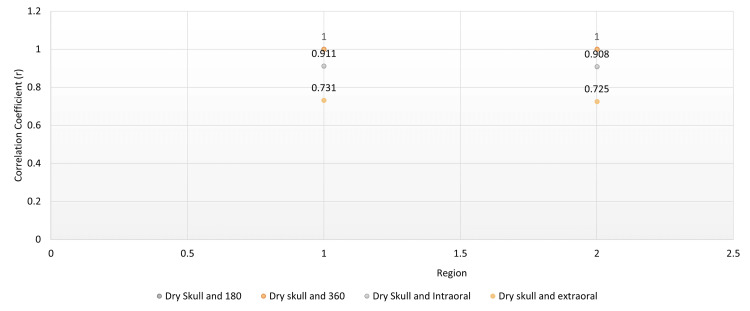
Agreement Analysis for Simulated Carious Defects

Comparison of intraoral and extraoral radiographs revealed one false positive and one false negative in intraoral radiographs, and two false positives and two false negatives in extraoral radiographs. The agreement measurements for all four imaging modalities against the simulated dry skull defects are described below (Figure 8). Overall, the kappa values indicated no statistically significant agreement.

The Friedman's test was used to assess differences in carious lesion depth measurements across imaging methods. The results showed no statistically significant differences, with p-values of 0.565 for mesial surfaces and 0.519 for distal surfaces.

For periodontal evaluation, a total of 180 sites - buccal, lingual, mesial, and distal surfaces of 45 teeth - were assessed. Simulated periodontal defects with bone loss greater than 2 mm were created at 137 sites, while 43 sites showed no defects, with bone loss measuring less than 2 mm. Intraoral and extraoral radiographs cannot depict the buccal and lingual measurements; therefore, only mesial and distal bone loss values were measured by these two modalities. However, the presence and absence of bone loss were evaluated on these radiographs.

**Figure 9:**
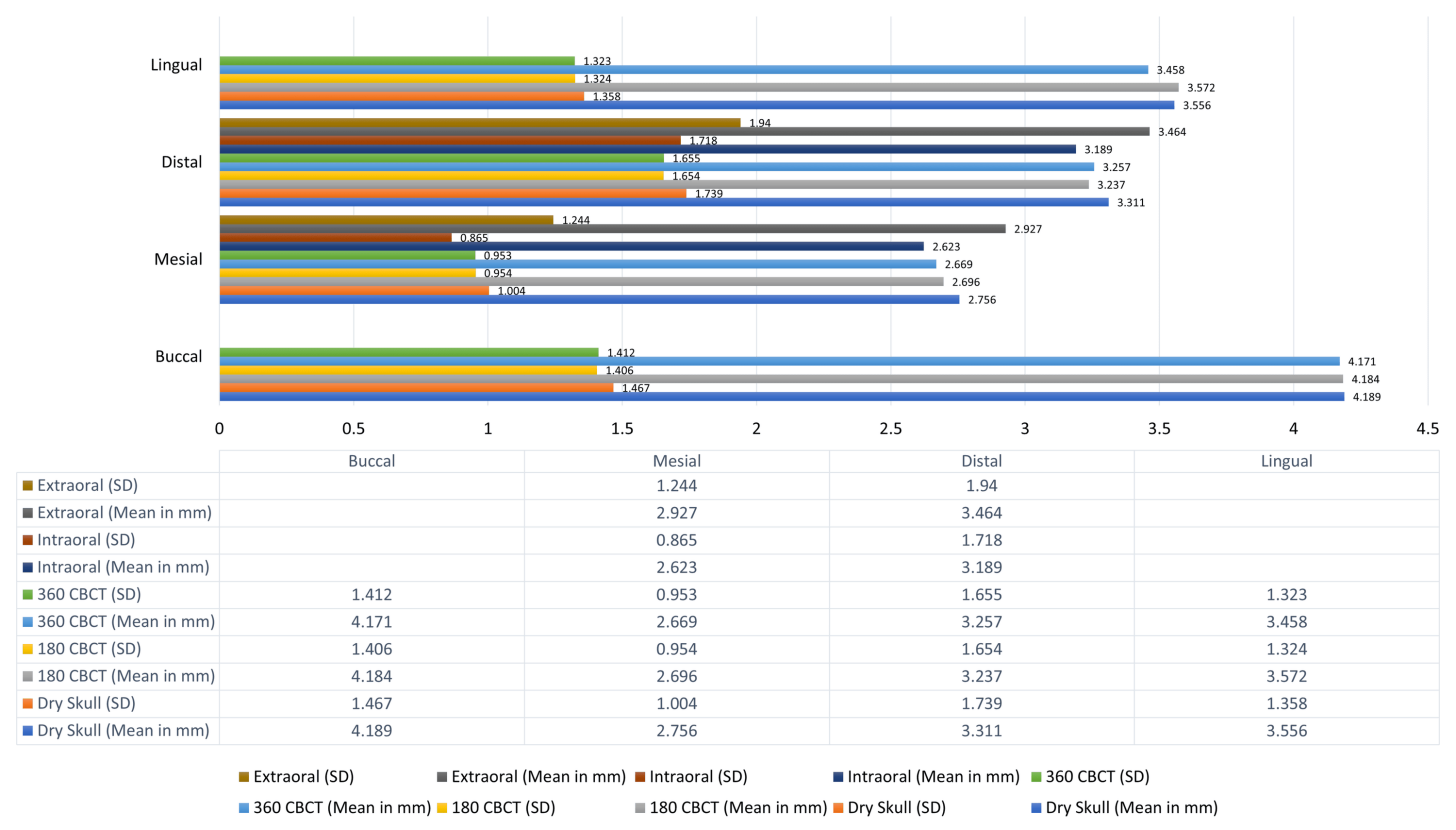
Comparison of mean bone loss measurements by different radiographic modalities CBCT: cone beam computed tomography

When compared to the mean values of simulated bone loss defects on the dry human mandible, the imaging modalities showed comparable results on the mesial and distal aspects. Similarly, the mean measurements on the buccal and lingual surfaces of the dry human skull were comparable to those obtained from CBCT scans with both 180- and 360-degree rotations. This also signifies that the values of 180- and 360-degree CBCT rotation scans are comparable amongst themselves as shown below (Figure 9).

Comparison of bone loss measurements across all radiographic modalities revealed no statistically significant differences, with p-values exceeding 0.001. This indicates that linear measurements were comparable across modalities, as shown in Table 3. All values were highly specific and calculated with a 99% confidence interval.

**Table 3:** Comparison of simulated periodontal bone loss defect measurements in four different modalities CBCT: cone beam computed tomography

Comparison	Buccal		Mesial		Distal		Lingual	
	‘t’ value	P value	‘t’ value	P value	‘t’ value	P value	‘t’ value	P value
Dry skull vs 180 CBCT	0.134	0.894	1.950	0.058	2.126	0.039	0.571	0.570
Dry Skull vs 360 CBCT	0.897	0.375	1.809	0.077	1.330	0.190	1.583	0.121
Dry skull vs Intraoral	-	-	2.130	0.039	2.399	0.021	-	-
Dry skull vs Extraoral	-	-	1.692	0.098	1.471	0.148	-	-

Eight interdental vertical periodontal defects were also evaluated based on the scoring criteria which include buccal wall in four defects and lingual wall in four defects. Lingual defects were not visible on intraoral and extraoral imaging, whereas all buccal defects were clearly visualized. Both 180-degree and 360-degree CBCT rotations demonstrated 100% accuracy in identifying the type of defect and the number of osseous walls present.

**Figure 10:**
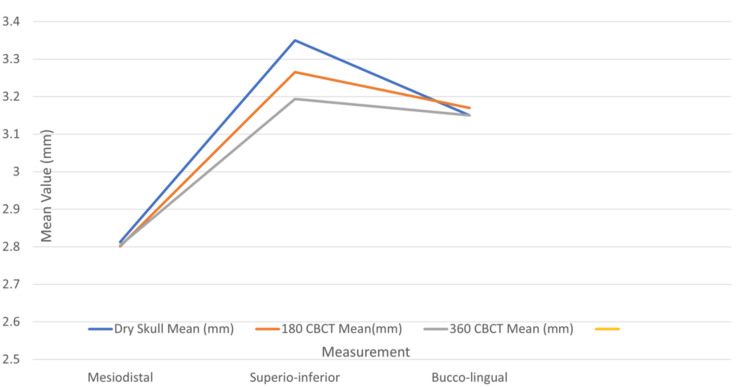
Comparison of the depth of defects in different directions in 180- and 360-generated bitewings CBCT: cone beam computed tomography

Furthermore, there was no statistical difference between the measurements obtained from the dry skull and CBCT-generated bitewings at 180- and 360-degree rotation when compared to the depth of the defects as the p-value is more than 0.05 as shown below (Figure 10).

## Discussion

The 2016 Global Burden of Disease Study reported that oral health issues, especially dental caries in permanent teeth, impacted nearly half of the global population [8]. In light of the COVID-19 pandemic, there has been a growing emphasis on diagnostic approaches that reduce salivary aerosol exposure and minimize the need for extensive personal protective equipment. Although clinical examination remains valuable in detecting various carious lesions, it is not always effective in identifying early-stage proximal caries that are not directly visible. Therefore, bitewing radiographs remain an essential tool for diagnosing approximal carious lesions.

Intraoral digital imaging poses specific clinical challenges, particularly for patients who have a pronounced gag reflex or restricted mouth opening [9]. Factors such as trismus from impacted third molars, a high floor of the mouth, soft tissue enlargements, or other head and neck abnormalities may hinder the proper placement of intraoral detectors. In such scenarios, extraoral imaging presents a valuable alternative, requiring less patient compliance and lowering the risk of cross-contamination - an advantage that became especially significant during the COVID-19 pandemic. Research has explored the utility of extraoral radiographs in simultaneously assessing caries and periodontal health, and their clinical value has been further highlighted in situations demanding reduced contact and improved infection control.

Panoramic radiography provides a comprehensive overview and, when configured to produce bitewing-like images, can effectively detect both dental caries and periodontal conditions in a single scan. This technique overcomes the limitations of intraoral sensor placement and enhances the efficiency of image acquisition.

Our study revealed that there were no notable differences in the ability to detect interproximal caries - regardless of lesion depth - when comparing intraoral bitewings, panoramic-based bitewings, and those generated using CBCT. These outcomes align with the findings of Terry et al. (2016), who also investigated and confirmed the diagnostic effectiveness of multiple radiographic techniques for identifying approximal carious lesions [10].

Nevertheless, two-dimensional imaging methods, whether intraoral or extraoral, do not capture buccolingual bone dimensions. In contrast, CBCT offers three-dimensional visualization, allowing clinicians to detect defects that might be hidden due to superimposed anatomical structures, making it especially useful in complex diagnostic situations.

CBCT offers superior spatial resolution with a lower radiation dose compared to conventional multislice CT scans [11]. Its accuracy in visualizing dental and periodontal structures has been validated in multiple studies [12-18]. There are no studies to compare the diagnostic efficacy of all three modalities in the detection of carious defects, bone loss measurements, and simulated osseous defects which was the knowledge gap and one of the aims of our study.

Image quality in CBCT is influenced by factors such as kVp, mA, voxel size, and FOV [19]. To access the periodontal structures, such as the periodontal ligament space, cortical bone, alveolar crest, and cortical plates, images with improved resolution are needed as well as a smaller voxel size. Keeping that in mind, CBCT scans were obtained using a high-resolution protocol (90 kVp, 7 mA) at both 180° and 360° rotations with a voxel size of 80 μm. The high-resolution mode improves image clarity by increasing the number of basis images, although it comes with a slightly increased radiation dose due to prolonged scanning time.

Artifacts from metallic restorations, which can compromise CBCT image quality, were controlled by excluding affected samples from the study.

In assessing periodontal health, bone loss was defined as a distance greater than 2 mm from the CEJ to the alveolar crest (AC), in alignment with common clinical standards [20-23]. The measurements of linear bone loss showed no statistically significant difference across the imaging modalities in the current study.

Angular osseous defects were reliably identified exclusively through CBCT images. While 2D bitewings failed to visualize defects involving the lingual plate, CBCT imaging - both 180° and 360° - accurately captured defects involving both buccal and lingual walls. Moreover, there were no significant differences in defect width and depth measurements between the two CBCT acquisition angles, with results aligning closely with dry skull references and findings from studies by Misch et al. [18] and Vasconcelos et al. [24].

These results reinforce that while 2D imaging is adequate for routine interproximal bone loss detection, CBCT provides added diagnostic value in identifying complex periodontal defects, especially vertical defects with multiple osseous walls. This is crucial for surgical and regenerative periodontal procedures, where detailed anatomical insight significantly enhances treatment planning. Our results were supported by the study conducted by Mol et al. and Misch et al. [17,18].

Although 180° CBCT rotations reduce patient dose by capturing fewer basis images, this can compromise image resolution. Therefore, adhering to the As Low As Reasonably Achievable (ALARA) principle remains essential when selecting imaging protocols.

One limitation of our study was the use of a circular collimator, which results in higher radiation exposure compared to rectangular collimators. However, circular collimation was chosen to minimize retakes, a common issue with rectangular collimation. Additionally, we used a round bur to simulate carious lesions, as opposed to acid etching, to ensure standardized lesion depth and control over defect dimensions.

## Conclusions

Extraoral bitewings generated from panoramic radiographs and CBCT (180° and 360°) scans demonstrated diagnostic accuracy comparable to intraoral bitewings for the detection of approximal caries and periodontal bone defects.

## References

[1] (2020). Nationwide Evaluation of X-ray Trends (NEXT): tabulation and graphical summary of the 2014-2015 survey of dental facilities. https://crcpd.org/wp-content/uploads/2023/11/E-19-2_2014-2015_Dental_NEXT_Summary_Report.pdf.

[2] Riaud X (2018). First dental radiograph(1896). J Dent Health Oral Disord Ther.

[3] Johnson KB, Mol A, Tyndall DA (2021). Extraoral bite-wing radiographs: a universally accepted paradox. J Am Dent Assoc.

[4] Abdinian M, Razavi SM, Faghihian R (2015). Accuracy of digital bitewing radiography versus different views of digital panoramic radiography for detection of proximal caries. J Dent (Tehran).

[5] Abu El-Ela WH, Farid MM, Mostafa MS (2016). Intraoral versus extraoral bitewing radiography in detection of enamel proximal caries: an ex vivo study. Dentomaxillofac Radiol.

[6] Clifton TL, Tyndall DA, Ludlow JB (1998). Extraoral radiographic imaging of primary caries. Dentomaxillofac Radiol.

[7] Kamburoglu K, Kolsuz E, Murat S, Yüksel S, Ozen T (2012). Proximal caries detection accuracy using intraoral bitewing radiography, extraoral bitewing radiography and panoramic radiography. Dentomaxillofac Radiol.

[8] Jin LJ, Lamster IB, Greenspan JS, Pitts NB, Scully C, Warnakulasuriya S (2016). Global burden of oral diseases: emerging concepts, management and interplay with systemic health. Oral Dis.

[9] Fratila AM, Saceleanu A, Arcas VC, Fratila N, Earar K (2025). Enhancing intraoral scanning accuracy: from the influencing factors to a procedural guideline. J Clin Med.

[10] Terry GL, Noujeim M, Langlais RP, Moore WS, Prihoda TJ (2016). A clinical comparison of extraoral panoramic and intraoral radiographic modalities for detecting proximal caries and visualizing open posterior interproximal contacts. Dentomaxillofac Radiol.

[11] Lechuga L, Weidlich GA (2016). Cone beam CT vs. fan beam CT: a comparison of image quality and dose delivered between two differing CT imaging modalities. Cureus.

[12] Frederiksen NL, Benson BW, Sokolowski TW (1994). Effective dose and risk assessment from film tomography used for dental implant diagnostics. Dentomaxillofac Radiol.

[13] Silva MA, Wolf U, Heinicke F, Bumann A, Visser H, Hirsch E (2008). Cone-beam computed tomography for routine orthodontic treatment planning: a radiation dose evaluation. Am J Orthod Dentofacial Orthop.

[14] Nakajima A, Sameshima GT, Arai Y (2005). Two, and three-dimensional orthodontic imaging using limited cone-beam computed tomography. Angle Orthod.

[15] Tsiklakis K, Syriopoulos K, Stamatakis HC (2004). Radiographic examination of the temporomandibular joint using cone beam computed tomography. Dentomaxillofac Radiol.

[16] Holberg C, Steinhäuser S, Geis P, Rudzki-Janson I (2005). Cone-beam computed tomography in orthodontics: benefits and limitations. J Orofac Orthop.

[17] Mol A, Balasundaram A (2008). In vitro cone beam computed tomography imaging of periodontal bone. Dentomaxillofac Radiol.

[18] Misch KA, Yi ES, Sarment DP (2006). Accuracy of cone beam computed tomography for periodontal defect measurements. J Periodontol.

[19] Lagos de Melo LP, Queiroz PM, Moreira-Souza L, Nadaes MR, Santaella GM, Oliveira ML, Freitas DQ (2023). Influence of CBCT parameters on image quality and the diagnosis of vertical root fractures in teeth with metallic posts: an ex vivo study. Restor Dent Endod.

[20] Wong BK, Leichter JW, Chandler NP, Cullinan MP, Holborow DW (2007). Radiographic study of ethnic variation in alveolar bone height among New Zealand dental students. J Periodontol.

[21] Lennon MA, Davies RM (1974). Prevalence and distribution of alveolar bone loss in a population of 15-year-old schoolchildren. J Clin Periodontol.

[22] Latcham NL, Powell RN, Jago JD, Seymour GJ, Aitken JF (1983). A radiographic study of chronic periodontitis in 15 year old Queensland children. J Clin Periodontol.

[23] Gargiulo AW, Wentz FM, Orban B (1961). Dimensions and relations of the dentogingival junctions in humans. J Periodontol.

[24] de Faria Vasconcelos K, Evangelista KM, Rodrigues CD, Estrela C, de Sousa TO, Silva MA (2012). Detection of periodontal bone loss using cone beam CT and intraoral radiography. Dentomaxillofac Radiol.

